# Inhibition of Mitochondrial Complex Function—The Hepatotoxicity Mechanism of Emodin Based on Quantitative Proteomic Analyses

**DOI:** 10.3390/cells8030263

**Published:** 2019-03-20

**Authors:** Longfei Lin, Yuling Liu, Sai Fu, Changhai Qu, Hui Li, Jian Ni

**Affiliations:** 1 Institute Chinese materia medica china academy of Chinese medical sciences, Beijing 100700, China; linlongfei0417@126.com (L.L.); ylliu@icmm.ac.cn (Y.L.); fusai920412@126.com (S.F.); 2 School of Chinese material medica, Beijing University of Chinese Medicine, Beijing 100102, China; quchanghai@bucm.edu.cn

**Keywords:** emodin, hepatotoxicity, proteomic, mitochondrial, complex

## Abstract

Emodin is the main component of traditional Chinese medicines including rhubarb, *Polygonum multiflorum*, and *Polygonum cuspidatum*. It has confirmed hepatotoxicity and may be the main causative agent of liver damage associated with the above-mentioned traditional Chinese medicines. However, current research does not explain the mechanism of emodin in hepatotoxicity. In this study, L02 cells were used as a model to study the mechanism of emodin-induced hepatocyte apoptosis using quantitative proteomics, and the results were verified by Western blot. A total of 662 differentially expressed proteins were discovered and analyzed using Gene Ontology (GO) and pathway enrichment analysis. The results show that the oxidative phosphorylation pathway is highly represented. Abnormalities in this pathway result in impaired mitochondrial function and represent mitochondrial damage. This result is consistent with mitochondria membrane potential measurements. Analysis of differentially expressed proteins revealed that emodin mainly affects oxidative phosphorylation pathways by inhibiting the function of the mitochondrial respiratory chain complexes; the mitochondrial respiratory chain complex activity assay result also confirmed that emodin could inhibit the activity of all mitochondrial complexes. This results in an increase in caspase-3, a decrease in mitochondrial membrane potential (MMP,) an increase in reactive oxygen species (ROS), and disorders in ATP synthesis, etc., eventually leading to mitochondrial damage and hepatocyte apoptosis in vitro.

## 1. Introduction

Emodin is an anthraquinone mainly found in traditional Chinese medicines such as rhubarb, *Polygonum multiflorum*, and *Polygonum cuspidatum*. It has many pharmacological activities, such as anti-cancer and anti-inflammatory effects, constipation relief, liver protection, and anti-aging effects [1,2,3,4,5,6]. Emodin has a high content in those plants mentioned above, reaching close to 1% in *Polygonum cuspidatum* and *Polygonum multiflorum* [7,8,9,10]. The daily dose of *Polygonum cuspidatum* should be 9–15 g, as stipulated in the Chinese Pharmacopoeia, so the dose of emodin can be up to 150 mg when taken in *Polygonum cuspidatum*. Furthermore, in recent years, the liver damage caused by Chinese herbal medicines such as *P. multiflorum* and rhubarb [11,12,13,14,15] has attracted widespread attention. Many studies have shown that the main component, emodin, has strong hepatotoxicity and can cause liver damage [13,16,17]. The U.S. National Toxicology Program (NTP) verified this finding through a two-year study of emodin [18]. Previous in vitro studies have also shown that emodin can cause apoptosis in normal human L02 cells [13,16]. These studies employed high-content analysis, which confirmed that emodin-induced apoptosis of L02 cells is related to changes in mitochondrial membrane potential (MMP) and increased reactive oxygen species (ROS). However, there are currently no in-depth studies on the mechanism of action of emodin hepatotoxicity. Our study uses quantitative proteomic techniques to study the mechanism behind emodin-induced apoptosis in L02 cells and to verify these findings by Western blot. This study will allow us to better understand how liver damage can be caused by emodin and emodin-containing traditional Chinese medicines and to provide a reference for future research.

## 2. Materials and Methods

### 2.1. Materials

Normal human liver cell line L02 cells were purchased from China Cell Bank (Shanghai). Cells were cultured in complete Dulbecco’s Modified Eagle Medium(DMEM) medium containing 10% fetal bovine serum (FBS, *v*/*v*) and 100 U/mL streptomycin. The culture conditions were 37 °C under 5% CO_2_. Emodin was purchased from Shanghai Yuanye Bio-Technology Co., Ltd. (Shanghai, China). Reducing reagent, denaturing reagent, iodoacetamide, quenching reagent, dissolution buffer, and the Protein Assay Kit were purchased from Thermo Scientific (Thermo Fisher Scientific Corp., MA, USA). Carbamide and 3-((3-cholamidopropyl) dimethylammonio)-1-propanesulfonate) (CHAPS) were purchased from Bio-Rad Laboratories, Inc. (Hercules, CA, USA). Thiourea and bovine serum albumin were purchased from Sigma-Aldrich Corporation (St. Louis, MO, USA). Caspase-8, caspase-3, Ndufs1, Cox7A2, ATP6, SDHA, and Bax inhibitor 1 antibodies were purchased from Cell Signaling Technology (Danvers, MA, USA). The mitochondrial extraction kit was purchased from Solarbio Science & Technology Corporation (Beijing, China). The Electron transport chain Complex assay kits I, II, III, IV, and V were purchased from Nanjing Jiancheng Bioengineering Institute (Nanjing, China).

### 2.2. Cell Culture

L02 cells were cultured in DMEM complete medium containing 10% FBS in a cell culture incubator under 37 °C, 5% CO_2_, and saturated humidity. When the cells were grown to 80–90% confluence, they were treated with 0.25% trypsin solution, passaged, and cells in good condition with vigorous proliferation were used for subsequent tests.

### 2.3. CCK8 Cell Survival Assay

L02 cells in logarithmic growth phase were treated with 0.25% trypsin and adjusted to a cell density of 5 × 10^4^ cells/mL; 100 μL cells per well were seeded in a 96-well plate, and cultured at 37 °C under 5% CO_2_ and saturated humidity. After incubation for 24 h, 100 μL of each test solution were added to each well at different concentrations. The test was set up with a positive control group (5% DMSO group) and a negative control group (same volume of culture medium), and each group contained four repeats. After the cells were cultured for 24 h to 48 h, the culture medium was removed, and 100 μL of fresh complete medium and 10 μL of CCK-8 reagent were added. The plate was placed in the incubator for another 3.5 h and a microplate reader was used to analyze the cells. Absorbance (A) at 450 nm was measured and the survival rate and inhibition rate of L02 cells were calculated as follows:

% survival = [OD value (test well) − OD value (background well)]/[OD value (negative control well) − OD value (background well)] × 100. % inhibition = 100% − % survival.

(OD is an abbreviation of optical density)

### 2.4. Quantitative Proteomic Assays

#### 2.4.1. Protein Extraction and Quantification

L02 cells were cultured in DMEM medium containing 10% FBS, 100 U/mL penicillin, and 100 U/mL streptomycin in a 5% CO_2_ incubator (Osaka, Japan) at 37 °C. The cell culture medium was replaced once every three days. L02 cells in logarithmic phase were collected, and 2 × 10^5^ cells/well were inoculated in 6-well plates. Cells were incubated at 37 °C until the cell density reached 80%, then were treated with culture medium and emodin for 48 h. The cells were collected, centrifuged, and the cell pellet was retained. An appropriate amount of lysis buffer (7 M urea, 2 M thiourea, and 0.1% CHAPS) was added to the pellet, and the sample was vortexed, sonicated, and centrifuged at 14,000× *g* for 30 min. The supernatant was taken for protein quantification by Bovine Serum Albumin (BCA); the control group and the administered group samples were prepared in triplicate, respectively.

#### 2.4.2. Proteolysis and Tandem Mass Tag (TMT) Labeling

From each sample, 100 μg of protein were taken and placed in a centrifuge tube, and dissolution buffer was added to a final volume of 100 μL. Reducing reagent was then added, and the samples were allowed to incubate at 55 °C for 1 h. Iodoacetamide solution was added and the samples were incubated at room temperature for 30 min in the dark. Dissolution buffer was added, following which the samples were centrifuged at 12,000 rpm for 20 min and solution was collected from the inner tube of the annular tubes (discard the solution in the outer tube). This step was repeated four times. The total volume was completed to 100 μL with dissolution buffer and 2 μg of trypsin were added to each sample (100 μg), which was allowed to incubate at 37 °C overnight. The tube was washed three times with ultrapure water, freeze-dried, and reconstituted with dissolution buffer.

TMT reagent was taken and 41 μL of absolute ethanol was added to each tube, which was then added to the enzyme-treated sample and allowed to react at room temperature for 1 h. Eight microliters of 5% quenching reagent was added and the samples were incubated for 15 min to stop the reaction. The labeled samples were mixed, vortexed, and centrifuged to the bottom of the tube. They were then vacuum-dried and frozen until use.

#### 2.4.3. LC/MS/MS Analysis

Reverse-phase chromatography at high pH: The mixed-labeled sample was dissolved in 100 μL of mobile phase A (98% ddH_2_O, 2% acetonitrile, pH 10), centrifuged at 14,000× *g* for 20 min, and the supernatant was taken for analysis. Separation was carried out using 400 μg of enzymatically digested bovine serum albumin (BSA). The column temperature was 45 °C, the detection wavelength was 214 nm, and 100 μL sample was loaded.

Nano-LC-Q exactive protein analysis: the components obtained by high pH reverse phase separation were reconstituted with 20 μL of 2% methanol and 0.1% formic acid, and centrifuged at 12,000 rpm for 10 min. The supernatant was aspirated and 10 μL were loaded using the sandwich method. The loading pump flow rate was 350 nl/min, for 15 min (mass spectrometry parameters).

#### 2.4.4. Analysis of Proteomics Data

The mass spectrometric analysis of TMT was performed by Thermo Q-Exactive mass spectrometry. The original mass spectrometry data generated were processed using Thermo’s commercial software Proteome Discoverer 2.1. The databases used were the UniProt and Homo sapiens databases. Using bioinformatics analysis tools, differentially expressed proteins were subjected to GO-based enrichment analysis (BP, cellular component (CC), and MF), and pathway enrichment analysis was performed using the KEGG database.

### 2.5. Western Blot Analysis

Total protein was extracted using the same method as described in Section 2.4.1, and a 6% stacking gel was poured onto a 10% SDS-polyacrylamide gel. After samples were loaded, electrophoresis was carried out and the gel was transferred to a polyvinylidene fluoride (PVDF) membrane. After blocking with 50 g/L skim milk for 1 h, the following primary antibodies were used: rabbit anti-human caspase-8, caspase-3, Ndufs1, Cox7A2, ATP6, SDHA, Bax inhibitor 1 protein, and anti-GAPDH antibody (1:1000 dilution). The secondary antibody was horseradish peroxidase-labeled goat anti-rabbit antibody (1:5000 dilution). The membrane was washed three times with phosphate tween buffer and the chemiluminescence reagent was developed. Each sample was run three times. After color development, the intensity of the bands was analyzed for semi-quantitative comparative analysis. The expression level of the protein was expressed as the ratio of the target protein intensity to the intensity of GAPDH.

### 2.6. MMP Measurement

Tetraethylbenzimidazolylcarbocyanine iodide (JC-1) is a widely used fluorescent probe for the measurement of MMP. When the MMP is high, the polymer emits red fluorescence. When the MMP is low, JC-1 takes on a monomeric form that emits green fluorescence. The positive control was the mitochondrial uncoupler carbonyl cyanide m-chlorophenyl hydrazone (CCCP). JC-1 was diluted with ultrapure water, and then JC-1 staining buffer was added and mixed to make the JC-1 working solution. The control group, drug administration group, and vehicle group were cultured, and CCCP (10 mM) was added to the sample at a ratio of 1:1000 and incubated for 20 min. The JC-1 monomer was measured by the microplate reader at excitation/emission wavelengths of 490/530 nm, while the JC-1 polymer was measured at excitation/emission wavelengths of 525/590 nm. 

### 2.7. Mitochondrial Respiratory Chain Complexes Activity Assay

L02 cells were cultured in DMEM medium containing 10% FBS, 100 U/mL penicillin, and 100 U/mL streptomycin in a 5% CO_2_ incubator at 37 °C. L02 cells in logarithmic phase were collected, and 2 × 10^5^ cells/well were inoculated in 6-well plates. Cells were incubated at 37 °C until the cell density reached 80%, then treated with culture medium and emodin at 50 μΜ for 48 h. After treatment the cells were collected, centrifuged, and the cell pellet was retained. An appropriate amount of lysis buffer was added for suspended cell, and then the mitochondria were extracted according to the instructions, and 5 × 10^7^ cells per time. After that, the activities of mitochondrial respiratory chain complexes I, II, III, IV, and V were quantitatively detected through the colorimetric method; the control group and the administered group samples were prepared in triplicate, respectively.

The activity values of the complexes are calculated as follows: 

Activity value = [(test sample OD value − background OD value) × system volume × test sample dilution multiple]/[test sample volume × X (millimole absorptivity) × reaction time(min) × test sample protein concentration (mg prot/mL)]

Different complexes have different X values, these being 5.5, 21.8, 21.84, 21.84, and 6.22 for complexes I, II, III, IV, and V, respectively. Prot is an abbreviation of protein.

## 3. Results

### 3.1. Cell Counting Kit-8(CCK-8) Analysis

**Figure 1:**
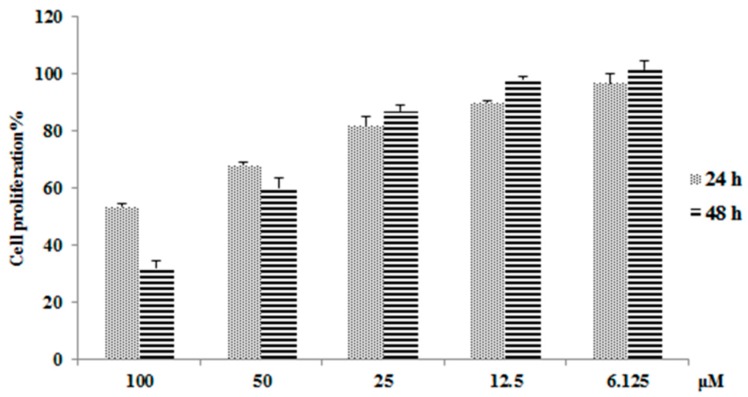
Cell proliferation results by Cell Counting Kit-8 (CCK-8) assay after emodin administrated for 24 h and 48 h (means ± SD, *n* = 3).

As shown in Figure 1, emodin has a strong ability to inhibit hepatocyte proliferation, inhibiting 46.7% of L02 cell growth (after 24 h) and 67.9% of L02 cell growth (after 48 h) at a concentration of 100 μM. This result is consistent with previous studies that showed that emodin has strong hepatotoxicity [16]. To further study the mechanism of action of emodin-induced hepatotoxicity, we planned to use proteomic analysis tools. To ensure the accuracy and consistency of our proteomics research, dose selection mainly refers to the concentration of emodin in plasma and liver after oral administration of *Polygonum cuspidatum* or other herbs which contain emodin. Studies have shown that a concentration of emodin up to 50 µM indicates a mitochondrial respiratory equivalent of 20 μM in plasma and 12 μg/g (approximately 27.8 μM, when considering the volume of 1 g liver tissue ≈ 1 mL) in liver after oral administration of *Polygonum cuspidatum* or rhubarb in rats, equal to the effective dose in humans [19,20]. Studies also shown that the plasma concentrations of emodin in diseased groups are higher than normal after administration [21]. Thus, the dose of emodin was kept to 50 μM in all samples, and the dosage time was 48 h. In addition, under these conditions, the hepatotoxic effect can be fully expressed while ensuring a sufficient number of viable cells required for downstream proteomic analyses. 

### 3.2. Protein Identification and Screening of Differentially Expressed Proteins

**Figure 2:**
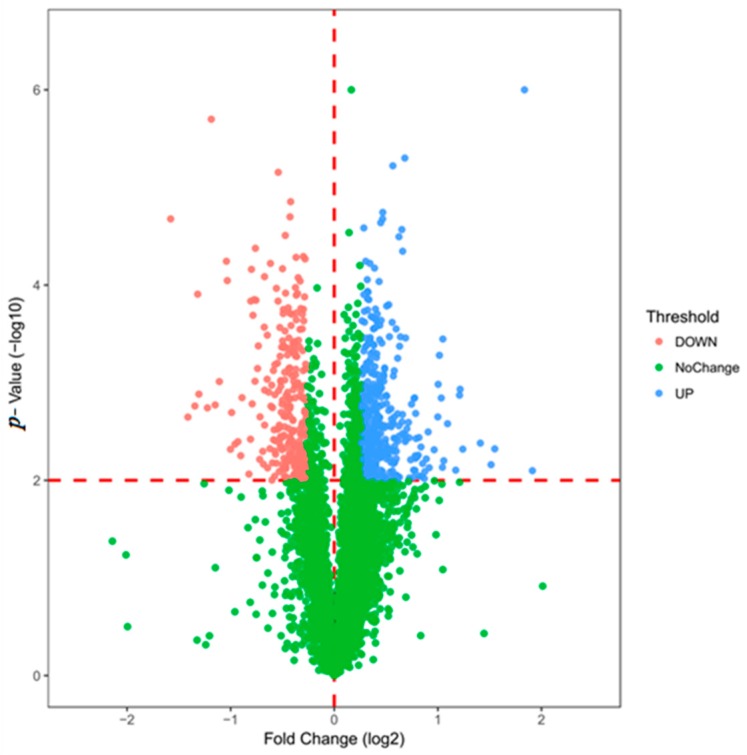
The differentially expressed proteins filter through a volcano map.

**Figure 3:**
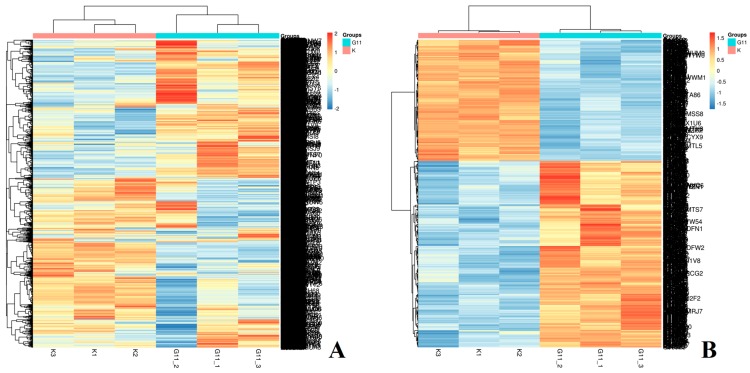
The hierarchical clustering of total protein (**A**) and differentially expressed proteins (**B**). * K1,2,3 are the three control groups; G11-1, -2, -3 are the three administered groups (*n* = 3).

After data processing, a total of 5758 proteins were identified. The identification criteria for differentially expressed proteins are as follows: *p*-value <0.01 and fold change >1.2 times were considered to be significant differences. The differential protein volcano map is shown in Figure 2. After screening, there were 662 differentially expressed proteins, of which 401 were up-regulated and 261 were down-regulated (Detailed in supplementary data). The expression pattern clustering analysis between multiple samples was used to observe the up-regulation and down-regulation of different proteins when compared between different samples. Figure 3 shows the method used for hierarchical clustering of total protein and differentially expressed proteins. The standard used to calculate the clustering distance index D was Euclidean distance. The distance between each group was calculated by the full connection method. Each row in the cluster heat map represents one protein. Each column represents a sample group. In these, the different colors represent the degree of differential expression, where green to black to red indicates increasing levels of differential expression. The length of the Euclidean distance between samples reflects their degree of similarity. A closer distance indicates that the data from the two groups are more similar, and a longer distance indicates that the data are less similar. Visually, the total protein and the differentially expressed proteins from the drug administration group and the control group show good clustering effects. The annotation on the right represents the significance of the difference in protein expression, which was generated by log2 conversion by the fold multiple. Red indicates an increase and blue indicates a decrease.

### 3.3. Gene Ontology (GO) and Pathway Enrichment Analysis

**Figure 4:**
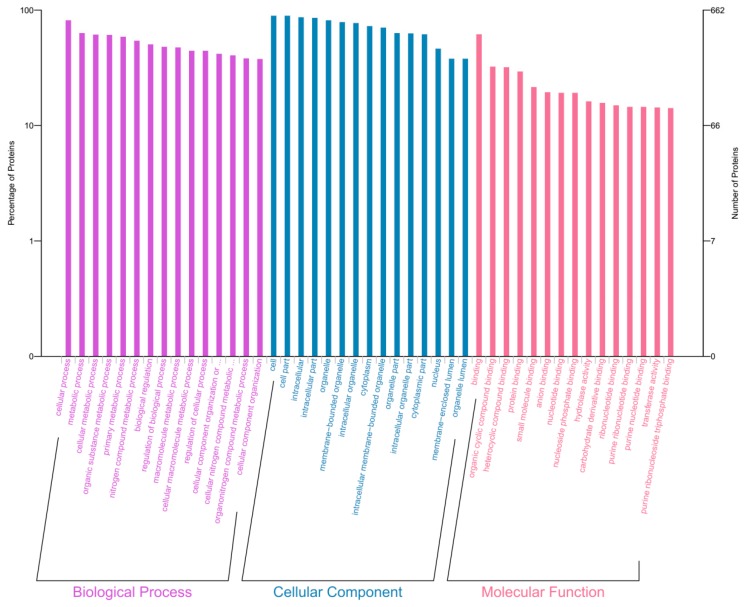
The results of Gene Ontology (GO) enrichment analysis of the differentially expressed proteins.

Differentially expressed proteins were analyzed using the Database for Annotation, Visualization and Integrated Discovery (DAVID) database and the results are shown in Figure 4. The biological processes (BPs) subcategory involved the cellular process, macromolecular complex subunit organization, and the cellular macromolecule metabolic process, etc. Molecular function (MF) represented the elemental activities of a gene product at the molecular level, such as binding or catalysis. For the analysis of MF, we found that most of the differentially expressed proteins were enriched in protein binding, small molecule binding, nucleoside phosphate binding, organic cyclic compound binding, and so on. For the analysis of the cellular component (CC), we found that most of the differentially expressed proteins were associated with the intracellular part, membrane-bounded organelle, organelle, cytoplasm, etc. Since mitochondrial function is closely related to apoptosis, mitochondria-related functions were further screened and the results are shown in Table 1. Clearly, emodin can cause a variety of mitochondrial dysfunctions, especially as these relate to mitochondrial respiratory chain functions, including the assembly and biosynthesis of mitochondrial respiratory chain complex I.

**Figure 5:**
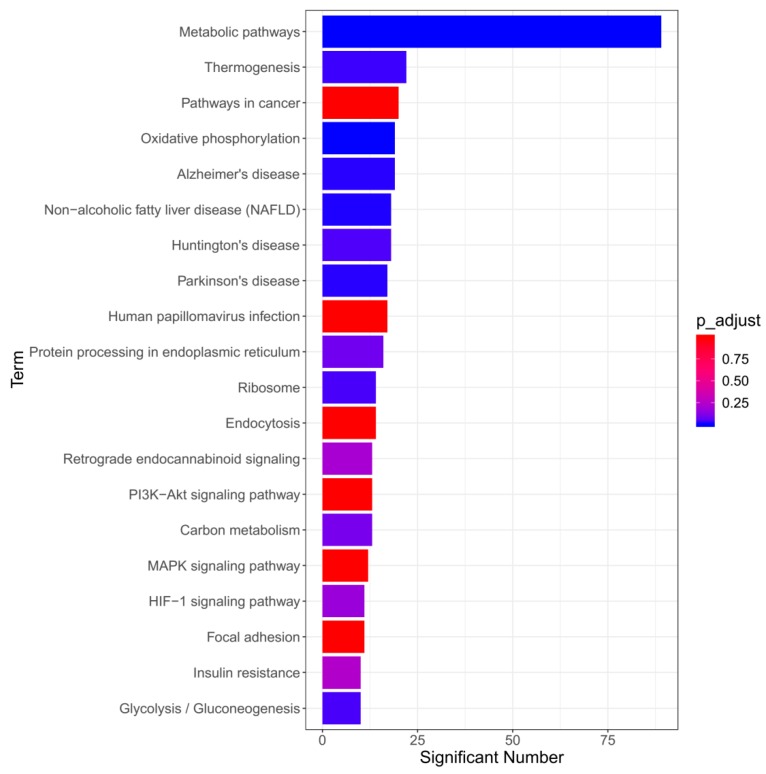
The results of pathway analysis of the differentially expressed proteins.

In order to explore the signaling pathways through which emodin promotes the development of liver injury, the differentially expressed proteins were analyzed using the DAVID and Kyoto Encyclopedia of Genes and Genomes (KEGG) databases, and the pathways associated with emodin are shown in Figure 5. These mainly include oxidative phosphorylation, non-alcoholic fatty liver disease (NAFLD), Alzheimer’s disease, Parkinson’s disease, Huntington’s disease, etc. Interestingly, all of the identified pathways are signaling pathways that are closely related to the mitochondrial respiratory chain and the respiratory chain complex. The above results indicate that hepatocyte apoptosis induced by emodin may be related to mitochondrial respiratory chain dysfunction.

### 3.4. Western Blot Analysis

**Figure 6:**
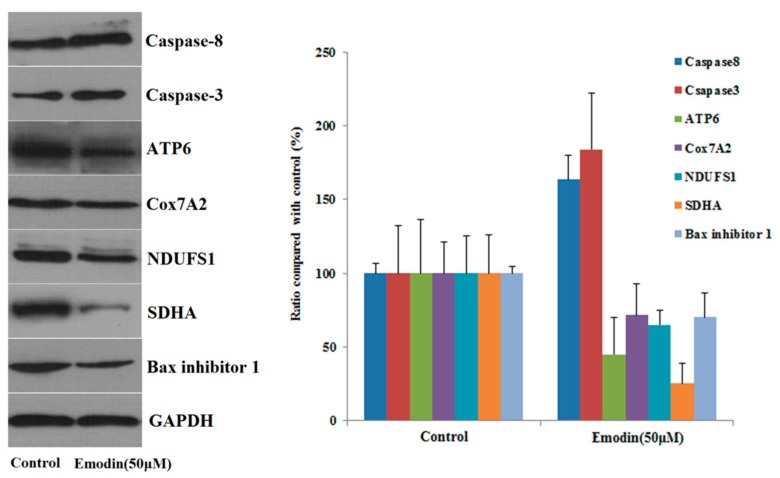
The expression of seven protein in L02 cells after treated with emodin for 48 h, (means ± SD, *n* = 3).

Western blotting was used to verify the accuracy of quantitative proteomic results and to further investigate the mechanism of emodin-induced apoptosis. The results of Western blot analysis of seven proteins show that the expression of caspase-8 increased after L-02 cells were treated with emodin, while the expression of Ndufs1, Cox7A2, ATP6, succinate dehydrogenase complex flavoprotein subunit A (SDHA), and Bax inhibitor 1 decreased. These findings are consistent with our proteomic analysis results, which indicate the reliability of our proteomics data. In addition, the increased expression of caspase-3 also indicates that the apoptotic pathway is activated with emodin treatment; the Western blot analysis results are shown in Figure 6.

### 3.5. MMP and Complex Activity Measurement

**Figure 7:**
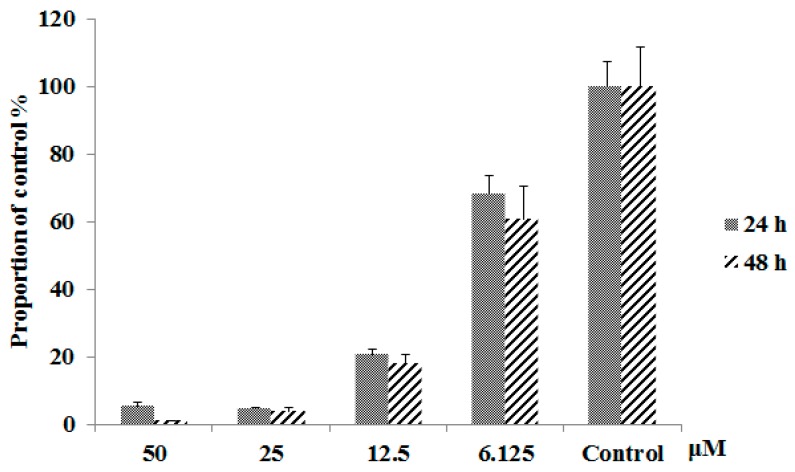
The changes of mitochondrial membrane potential (MMP) in L02 cells after treatment with emodin for 24 h and 48 h under different concentrations, (means ± SD, *n* = 3).

**Figure 8:**
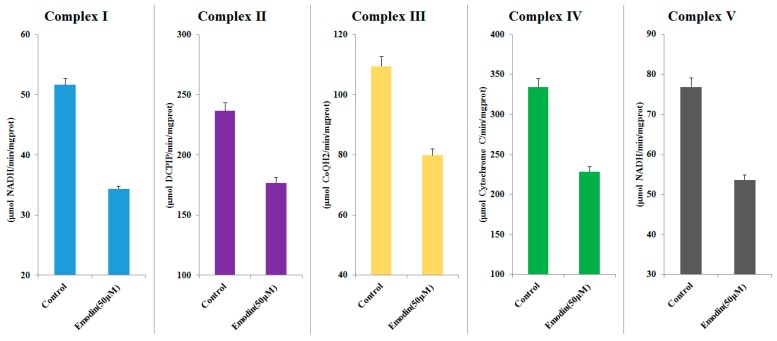
The results of mitochondrial respiratory chain complex activity assay (means ± SD, *n* = 3).

As shown in Figure 7, emodin has a significant influence on MMP. At concentrations of 25 μM and 50 μM, the MMP basically disappeared regardless of duration of drug treatment (24 h or 48 h). Even lower concentrations of emodin (e.g., 6.125 μM) cause a significant downward trend in MMP. The mitochondrial respiratory chain complex activity assay results are shown in Figure 8. The activity of all five complexes was reduced significantly after treatment with emodin at concentrations of 50 μM; as compared with control group, it was decreased by 30–40%. This result indicates that emodin could inhibit the activity of mitochondrial complexes, leading to oxidative phosphorylation pathway abnormality. This result is corroborated by the results of proteomics, and also indicates that the hepatotoxicity of emodin is indeed related to mitochondrial dysfunction.

## 4. Discussion

Mitochondria are abundant in mammalian liver cells and they are involved in a series of cellular physiological activities, including the regulation of electrolyte homeostasis in hepatocytes, ion transmembrane transport, oxygen free radical generation, cell signal transduction, and apoptosis [22,23,24]. Eighty percent of the energy required for cell life activities is provided by mitochondria; they are also the main organelles of cells for biological oxidation and energy conversion. Meanwhile, mitochondria are also among the most vulnerable organelles and can be indicators of the extent of cellular damage. A growing number of studies show that mitochondrial damage may be a predisposing factor and pathway for drug-induced liver injury [25]. Existing studies show that emodin or some anthraquinones can inhibit proton transport and induce activation of the mitochondrial apoptotic pathway, leading to liver damage [26,27,28,29].

Through proteomic analyses, we have shown that emodin-associated hepatotoxicity is associated with oxidative phosphorylation pathways, and our Western blots have confirmed the reliability of our proteomics results. Oxidative phosphorylation refers to the process by which electrons are transferred from NADH or FADH_2_ to molecular oxygen, producing water via an electron transport chain; this is coupled to the process that combines ADP with Pi to form ATP and is the main pathway for the synthesis of ATP. Inhibition of mitochondrial respiratory chain complex activity will lead to severe mitochondrial dysfunction. The results of complex activity measurement indicated that emodin affects expression of respiratory chain subunits and thus results in lower capacities. Furthermore, the results of MMP assays also show that the MMP of L02 cells is significantly decreased after emodin administration. Therefore, it is possible to speculate that energy deficiency and defective MMP could cause apoptosis [30,31,32].

Through KEGG pathway analysis, we can see that emodin mainly induces abnormalities in the oxidative phosphorylation pathway by affecting the activity of the respiratory chain complex. As shown in Table 2, emodin induces abnormalities in 20 subunits from four distinct complexes, of which 19 were shown to be abnormalities that are inhibitory to the function of the subunits. The most important of these is the inhibition of the activity of mitochondrial complex I, in which emodin inhibits the activity of 12 subunits in total. Mitochondrial complex I is the largest multi-subunit complex in the respiratory chain, and mutations that can lead to pathogenesis occur most frequently in this complex. Mitochondria are an important source of ROS, and complex I produces ROS through reverse electron transport. Inhibition of mitochondrial complexes leads to destruction of the electron transport chain and leads to a decrease in the production of intracellular ubiquinone, which in turn leads to an increase in ROS [33]. 

ROS can also cause the mitochondrial permeability transition pore (MPTP) to open by oxidizing two redox-sensitive sites on the MPTP, causing changes in mitochondrial membrane permeability [34]. In addition, the MMP established by the substrates of complex I and II is destroyed, which also leads to a decrease in transmembrane potential and promotes the opening of MPTP. The MPTP opening leads to the release of cytochrome C (Cyt C) into the matrix and Cyt C activates the apoptotic factor aspartate-specific caspase [35], which in turn causes cytotoxicity and leads to apoptosis. In addition, the release of Cyt C causes the electron transport chain to be blocked at Cyt C oxidase, leading to electron leaks and producing more ROS. Furthermore, the decrease or disappearance of MMP induces uncoupling in oxidative phosphorylation, thereby inhibiting the phosphorylation of ADP for the production of ATP, thus affecting mitochondrial energy metabolism [36,37,38].

Cytochrome oxidase (COX) is a key enzyme in the mitochondrial respiratory chain and the oxidative phosphorylation process and is also a major component of complex IV, which affects ATP synthesis and ROS production. COX has 13 subunits and mitochondrial DNA (mtDNA) encodes the largest of these, subunits I, II, and III. When mitochondria are damaged by oxidation, mtDNA undergoes structural changes such as fragment deletion, and this change in mtDNA is counterproductive to the production of COX [39,40], leading to errors in the assembly of COX I, II, III, and ATPase subunits of the respiratory chain. These errors in turn lead to a decrease in ATP and cause cell damage [40]. In addition, COX7A is a protein subunit that functions in complex IV. The abnormal expression of this protein subunit directly impedes the formation of respiratory complexes, causing functional defects of mitochondria and even cancer [41]. Besides, the result of proteomics and Western blot showed that the protein levels of SDHA in the administered group were significantly lower compared to those of the control group. SDHA encodes a major catalytic subunit of succinate-ubiquinone oxidoreductase, and it contains the flavin adenine dinucleotide (FAD) binding site where succinate is deprotonated and converted to fumarate. Abnormal SDHA expression was associated with mitochondrial respiratory chain deficiency. Studies have shown that decreased steady-state levels of SDHA will lead to impairment of complex II assembly and further cause mitochondrial respiratory chain disease [42,43].

**Figure 9:**
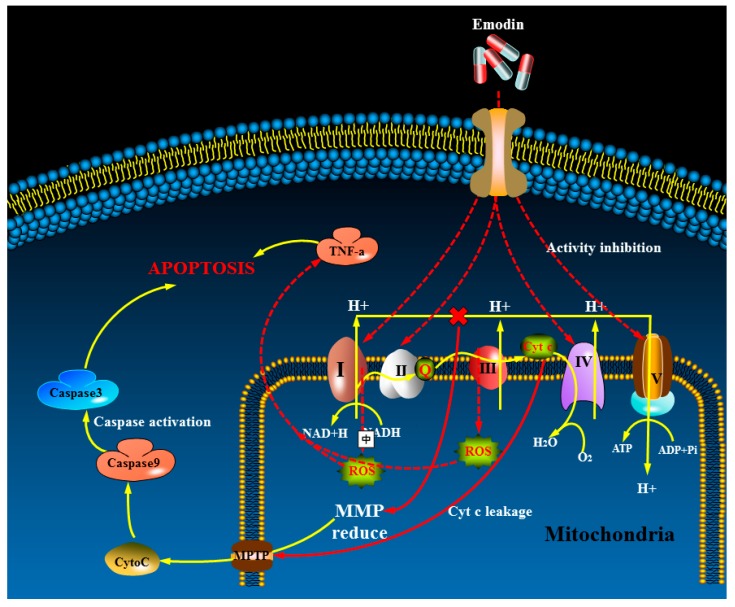
The possible hepatotoxicity signal pathway of apoptosis induced by emodin.

In conclusion, emodin-induced hepatocyte apoptosis mainly plays a role in the inhibition of the mitochondrial respiratory chain complex, then leading to mitochondrial dysfunction and energy deficiency. The mechanism of action is shown in Figure 9.

## 5. Conclusions

Emodin is a main component of rhubarb, *P. multiflorum*, and *P. cuspidatum*, and several studies have confirmed that it is related to the liver damage caused by these traditional Chinese medicinal herbs. In this study, quantitative proteomics were used to study the mechanism of action of L02 cell apoptosis caused by emodin in vitro. A total of 662 differentially expressed proteins were found. Through GO functional annotation, functional enrichment analysis, localization analysis, and pathway enrichment analysis, our results show that the oxidative phosphorylation pathway contributes highly to the mechanism of action of emodin, and abnormalities in the oxidative phosphorylation signaling pathway are representative of mitochondrial dysfunction and mitochondrial injury. The results of our study are consistent with those of other studies on the mechanism of liver injury caused by *P. multiflorum*. Further mitochondrial respiratory chain complex activity analysis revealed that emodin mainly inhibits the function of all mitochondrial respiratory chain complexes, leading to abnormalities in the oxidative phosphorylation pathway, a further reduction of MMP, and increasing ROS, leading to ATP synthesis disorders and eventually resulting in mitochondrial damage and apoptosis.

## Figures and Tables

**Table 1:** Representative results of GO enrichment analysis related to mitochondria functions.

GO.ID	Term	Annotated	Significant	*p*-Value	*p* Adjust	Names (Uniport Accession)	GO Category
GO:0098573	intrinsic component of mitochondrial membrane	86	6	9.4 × 10^−5^	1.12 × 10^−4^	A0A140TA86, P50416, Q16891, Q9Y3D6, P17152, Q99595	Cellular Component
GO:0032592	integral component of mitochondrial membrane	85	6	8.8 × 10^−5^	1.06 × 10^−4^	A0A140TA86, P50416, Q16891, Q9Y3D6, P17152, Q99595	Cellular Component
GO:0032543	mitochondrial translation	130	18	5.9 × 10^−16^	3.02 × 10^−15^	Q9BQC6, Q96EY7, Q9NYK5, Q9Y3D9, Q8N983, Q96A35, Q9BZE1, Q9H2W6, Q13084, O95182, Q8IXM3, Q969S9, Q9Y291, P82932, P82675, P46199, P42704, Q96DV4	Biological Process
GO:0140053	mitochondrial gene expression	147	18	5.3 × 10^−15^	2.33 × 10^−14^	Q9BQC6, Q96EY7, Q9NYK5, Q9Y3D9, Q8N983, Q96A35, Q9BZE1, Q9H2W6, Q13084, O95182, Q8IXM3, Q969S9, Q9Y291, P82932, P82675, P46199, P42704, Q96DV4	Biological Process
GO:0005747	mitochondrial respiratory chain complex I	75	11	4.3 × 10^−11^	1.29 × 10^−10^	P56556, O95182, O95139, O00217, Q16718, O00483, P28331, O43678, Q9NX14, O75306, P49821	Cellular Component
GO:0005746	mitochondrial respiratory chain	138	17	4.2 × 10^−15^	1.57 × 10^−14^	P56556, P31040, P13073, O95182, O95139, P21912, O00217, Q16718, O00483, H0UI06, P28331, O43678, P20674, Q7KZN9, Q9NX14, O75306, P49821	Cellular Component
GO:0033108	mitochondrial respiratory chain complex assembly	101	11	3.8 × 10^−9^	8.99 × 10^−9^	P56556, Q9NPL8, O95182, O95139, O00217, Q16718, P28331, O43678, Q9NX14, O75306, P49821	Biological Process
GO:0098800	inner mitochondrial membrane protein complex	400	20	3.6 × 10^−10^	9.53 × 10^−10^	P56556, A0A140TA86, Q9Y5J7, P31040, P13073, Q16891, O95182, O95139, P00846, P21912, O00217, Q16718, O00483, P28331, O43678, P20674, Q9NX14, O75306, Q99595, P49821	Cellular Component
GO:0006123	mitochondrial electron transport, cytochrome c to oxygen	20	4	3.5 × 10^−5^	3.9 × 10^−5^	P13073, O00483, P20674, Q7KZN9	Biological Process
GO:0098798	mitochondrial protein complex	430	22	3.0 × 10^−11^	9.43 × 10^−10^	O76031, P56556, A0A140TA86, Q9Y5J7, P31040, P13073, Q16891, O95182, O95139, P00846, F8W8Z9, P21912, O00217, Q16718, O00483, P28331, O43678, P20674, Q9NX14, O75306, Q99595, P49821	Cellular Component
GO:0006839	mitochondrial transport	316	14	2.6 × 10^−6^	3.88 × 10^−6^	Q9Y5J7, P50416, Q9Y3D6, O00170, Q9H300, P00846, Q14790, Q99436, P40763, Q5HYI7, O00165, Q9NQZ5, Q9BZL1, Q99595	Biological Process
GO:0070125	mitochondrial translational elongation	86	13	2.1 × 10^−12^	7.04 × 10^−12^	Q96EY7, Q9NYK5, Q9Y3D9, Q8N983, Q96A35, Q9BZE1, Q9H2W6, Q13084, Q8IXM3, Q9Y291, P82932, P82675, Q96DV4	Biological Process
GO:0032981	mitochondrial respiratory chain complex I assembly	62	11	1.7 × 10^−11^	5.13 × 10^−11^	P56556, Q9NPL8, O95182, O95139, O00217, Q16718, P28331, O43678, Q9NX14, O75306, P49821	Biological Process
GO:0097031	mitochondrial respiratory chain complex I biogenesis	62	11	1.7 × 10^−11^	5.13 × 10^−11^	P56556, Q9NPL8, O95182, O95139, O00217, Q16718, P28331, O43678, Q9NX14, O75306, P49821	Biological Process
GO:0005759	mitochondrial matrix	506	30	1.5 × 10^−16^	5.7 × 10^−16^	O76031, P48735, Q9BQC6, Q96RQ3, Q9UNQ2, Q07820, Q9NYK5, Q32P41, Q6NVY1, Q8N983, Q9BZE1, Q9H2W6, Q16822, Q13084, O95182, P30038, Q8IXM3, Q969S9, Q9Y291, P11498, Q5U5X0, Q8NFF5, P82932, O00217, P82675, P28331, O75306, P42704, Q96AG4, Q13057	Cellular Component
GO:0005761	mitochondrial ribosome	84	11	1.5 × 10^−10^	4.08 × 10^−10^	Q9BQC6, Q9NYK5, Q8N983, Q9BZE1, Q9H2W6, Q13084, O95182, Q8IXM3, Q9Y291, P82932, P82675	Cellular Component
GO:0005741	mitochondrial outer membrane	242	12	1.3 × 10^−6^	2.29 × 10^−6^	Q9NUQ2, Q07820, P50416, Q9Y3D6, Q969Z3, P00387, Q14790, F8W8Z9, Q5HYI7, O00165, Q13057, A0A0C4DFN1	Cellular Component
GO:0070126	mitochondrial translational termination	86	14	1.0 × 10^−13^	3.73 × 10^−13^	Q96EY7, Q9NYK5, Q9Y3D9, Q8N983, Q96A35, Q9BZE1, Q9H2W6, Q13084, Q8IXM3, Q969S9, Q9Y291, P82932, P82675, Q96DV4	Biological Process

**Table 2:** The differentially expressed proteins involved in the oxidative phosphorylation pathway and respiratory chain complex.

Accession	Description	Complex	Log Ratio (3/1)	*p*-Value
P28331	NADH:ubiquinone oxidoreductase core subunit S1 (NDUFS1)	Complex I	−0.7842	0.0002
O75306	NADH:ubiquinone oxidoreductase core subunit S2 (NDUFS2)		−0.4512	0.0017
O00217	NADH:ubiquinone oxidoreductase core subunit S8 (NDUFS8)		−0.3746	0.0005
P49821	NADH:ubiquinone oxidoreductase core subunit V1 (NDUFV1)		−0.5872	0.0008
E7EPT4	NADH:ubiquinone oxidoreductase core subunit V2 (NDUFV2)		−0.4440	0.0063
O43678	NADH:ubiquinone oxidoreductase subunit A2 (NDUFA2)		−0.2866	0.0001
Q16718	NADH:ubiquinone oxidoreductase subunit A5 (NDUFA5)		−0.4784	0.0028
P56556	NADH:ubiquinone oxidoreductase subunit A6 (NDUFA6)		−0.7647	0.0010
O95182	NADH:ubiquinone oxidoreductase subunit A7 (NDUFA7)		0.4626	0.0017
Q9NX14	NADH:ubiquinone oxidoreductase subunit B11 (NDUFB11)		−0.5076	0.0004
O95139	NADH:ubiquinone oxidoreductase subunit B6 (NDUFB6)		−0.3481	0.0001
O00483	NDUFA4, mitochondrial complex associated (NDUFA4)		−0.4872	0.0004
P31040	succinate dehydrogenase complex flavoprotein subunit A (SDHA)	Complex II	−0.7329	0.0004
P21912	succinate dehydrogenase complex iron sulfur subunit B (SDHB)		−0.5184	0.0046
Q7KZN9	COX15, cytochrome c oxidase assembly homolog (COX15)	Complex IV	−0.4991	0.0001
P13073	cytochrome c oxidase subunit 4I1 (COX4I1)		−0.3085	0.0003
P20674	cytochrome c oxidase subunit 5A (COX5A)		−0.5156	0.0011
H0UI06	cytochrome c oxidase subunit 7A2 (COX7A2)		−0.7194	0.0061
P00846	ATP synthase F0 subunit 6 (ATP6)	Complex V	−1.0014	0.0048
Q9Y487	ATPase H+ transporting V0 subunit a2 (ATP6V0A2)		0.3322	0.0043

## Supplementary Materials

The supplementary materials are available online at https://www.mdpi.com/2073-4409/8/3/263/s1.

## Abbreviations

BP	Biological processes
BSA	Bovine serum albumin
CC	Cellular component
CCCP	Carbonyl cyanide m-chlorophenyl hydrazine
CCK-8	Cell Counting Kit-8
CHAPS	3-((3-cholamidopropyl) dimethylammonio)-1-propanesulfonate)
COX	Cytochrome oxidase
Cyt C	Cytochrome C
DAVID	Database for Annotation, Visualization and Integrated Discovery
DMEM	Dulbecco’s Modified Eagle Medium
FAD	Flavin adenine dinucleotide
GO	Gene ontology
JC-1	Tetraethylbenzimidazolylcarbocyanine iodide
KEGG	Kyoto Encyclopedia of Genes and Genomes
MF	Molecular function
MMP	Mitochondrial membrane potential
MPTP	Mitochondrial permeability transition pore
mtDNA	Mitochondrial DNA
NADH	Nicotinamide adenine dinucleotide
NAFLD	Non-alcoholic fatty liver disease
NTP	National Toxicology Program
PVDF	Polyvinylidene fluoride
Q	Coenzyme Q
ROS	Reactive oxygen species
SDHA	Succinate dehydrogenase complex flavoprotein subunit A
TMT	Tandem Mass Tag
TNF-α	Tumor necrosis factor-α
